# Downregulation of PRKCI inhibits osteosarcoma cell growth by inactivating the Akt/mTOR signaling pathway

**DOI:** 10.3389/fonc.2024.1389136

**Published:** 2024-07-02

**Authors:** Liujing Qu, Yu Xin, Jieni Feng, Xiaolei Ren, Zuming Li, Xueru Chen, Guangyan Miao, Jiankun Chen, Chengming Sun, Yue Lu

**Affiliations:** ^1^ Department of Clinical Laboratory, The Affiliated Yantai Yuhuangding Hospital of Qingdao University, Yantai, China; ^2^ Department of Medical Laboratory, Qingdao Sixth People’s Hospital, Qingdao, China; ^3^ The Second Clinical Medical College, Guangzhou University of Chinese Medicine, Guangzhou, China; ^4^ Department of Molecular, Cell and Cancer Biology, University of Massachusetts Chan Medical School, Worcester, MA, United States; ^5^ The Third Comprehensive Department, The Second Affiliated Hospital of Guangzhou University of Chinese Medicine (Guangdong Provincial Hospital of Chinese Medicine), Guangzhou, China; ^6^ State Key Laboratory of Dampness Syndrome of Chinese Medicine, The Second Affiliated Hospital of Guangzhou University of Chinese Medicine (Guangdong Provincial Hospital of Chinese Medicine), Guangzhou, China; ^7^ Guangdong-Hong Kong-Macau Joint Lab on Chinese Medicine and Immune Disease Research, Guangzhou University of Chinese Medicine, Guangzhou, China

**Keywords:** PRKCI, osteosarcoma, proliferation, Akt/mTOR signaling pathway, therapy

## Abstract

PRKCI is abnormally expressed in various cancers, but its role in osteosarcoma is unknown. This study aimed to explore the biological function of PRKCI in osteosarcoma and its potential molecular mechanism. PRKCI expression was evaluated in osteosarcoma cell lines using Western blot analysis and reverse transcription PCR. The CCK-8 assay, colony formation assay, flow cytometry, Transwell assay, and wound-healing assay were used to detect the proliferation, colony-forming capacity, cell cycle, migration, and invasion of osteosarcoma cells when PRKCI was overexpressed or knocked down. The interaction between PRKCI and SQSTM1 was explored using immunoprecipitation. Finally, the protein molecule expression of the Akt/mTOR signaling pathway in osteosarcoma was detected when PRKCI was knocked down. Our study found that PRKCI was overexpressed in osteosarcoma cell lines. The overexpression of PRKCI promoted the proliferation and colony-forming capacity of osteosarcoma cells, while silencing PRKCI inhibited the proliferation, colony-forming capacity, migration, and invasion of osteosarcoma cells and arrested the cell cycle at the G2/M phase. Both PRKCI and SQSTM1 were overexpressed in osteosarcoma. The expression of PRKCI was only related to histological type, while that of SQSTM1 was not related to clinical characteristics. The expression of PRKCI and SQSTM1 in osteosarcoma was higher than that in chondrosarcoma. Knockdown of PRKCI inhibited the proliferation of osteosarcoma cells by inactivating the Akt/mTOR signaling pathway, suggesting that PRKCI was a potential target for osteosarcoma therapy.

## 1 Introduction

Osteosarcoma is the most common primary bone malignancy, especially in children and adolescents. Its annual incidence rate was 4.0 per million in the range of 0–14 years and 5.0 per million in the range of 0–19 years (1). Osteosarcoma, which originates from primitive mesenchymal bone-forming cells, is a common malignant tumor of the distal femur, proximal tibia, and proximal humerus and often invades and metastasizes to the lung (2). Surgery and chemotherapy greatly improved the survival rate of osteosarcoma in the 1970s and 1980s but had little effect on metastatic patients and recurrent patients (3, 4). Even worse, the survival rate of osteosarcoma patients has not improved in the past few decades (5). Therefore, exploring the potential molecular mechanism in osteosarcoma is of great significance in finding new therapeutic strategies and improving the prognosis of patients. Many studies have proposed that multiple molecules such as cyclin-dependent kinase 9, Wilms’ tumor 1-associated protein, and aurora B kinase are expected to become the therapeutic targets of osteosarcoma (6–8), indicating that targeted therapy against related molecules is a promising strategy.

PKCι (PRKCI) is a member of the gene family encoding serine/threonine kinase, belonging to the atypical protein kinase C family. According to previous reports, PRKCI is abnormally expressed in cancers of various organs and tissues, including the lung, stomach, colon, breast, bile duct, and prostate (9), but there are no data on osteosarcoma. Hina et al. found that PRKCI is involved in the occurrence of ovarian cancer and promotes the proliferation of ovarian cancer (10). Chen et al. pointed out that PRKCI promotes the viability, invasion, and migration of hepatocellular carcinoma cells by upregulating the expression level of FOXK1 (11). In addition, other studies have shown that PRKCI is related to autophagy and apoptosis (12, 13). However, the role of PRKCI in osteosarcoma cells remains to be determined.

SQSTM1, also known as sequestosome 1, is a protein involved in various cellular processes such as autophagy, apoptosis, inflammation, and cell survival. Accumulation of SQSTM1 reflects impaired autophagy, which is related to the carcinogenesis and progression of various tumors, including hepatocellular carcinoma (HCC) (14). The interaction between PRKCI and SQSTM1 regulates autophagy, thus playing a role in liver cancer development (13). However, their interaction in osteosarcoma is unclear.

Akt/mTOR signaling pathway is involved in a variety of human physiological and pathological processes such as cell proliferation, apoptosis, autophagy, development of neuronal dendrites, inflammation, and tumors (15–17). It is significantly upregulated in a variety of cancers and is pointed out to be closely related to the occurrence and progression of tumors (18). Several studies have found abnormal expression of the Akt/mTOR signaling pathway in osteosarcoma (19), but it is not known whether it is involved in the effect of PRKCI on osteosarcoma.

In this study, we detected the expression of PRKCI in osteosarcoma cell lines and observed its effects on osteosarcoma cell proliferation, cell cycle, migration, and invasion by upregulating or silencing the expression of PRKCI. Then, we detected the expression of PRKCI and SQSTM1 in human osteosarcoma tissues and analyzed their correlation with the clinical characteristics of osteosarcoma, respectively. Furthermore, we identified the interaction between PRKCI and SQSTM1 in osteosarcoma cell lines. Finally, we explored the relationship between PRKCI and the Akt/mTOR signaling pathway in osteosarcoma cell lines.

## 2 Materials and methods

### 2.1 Cell lines and cell culture

One osteosarcoma cell line (U2OS), one human osteoblast cell line (Saos2), and one human chondrosarcoma cell line (SW1353) were used in this study. They were gifts from Prof. Yingyu Chen (Peking University Health Science Center, China). All cell lines were cultured in Dulbecco’s modified Eagle’s medium (DMEM) supplemented with 10% fetal bovine serum (FBS) in a humidified atmosphere containing 5% CO_2_ at 37°C.

### 2.2 RNA extraction and reverse transcription PCR

Total RNA samples were extracted from cells with the TRIzol reagent (Invitrogen, 15596–026, Carlsbad, CA, USA) according to the manufacturer’s instructions. Reverse transcription PCR (RT-PCR) was performed using the ThermoScript RT-PCR System (Invitrogen, 11146–024, Carlsbad, CA, USA). Primers used for amplifying PRKCI were as follows: 5′-AGTGAGCCCACCTTAGAC-3′ (forward) and 5′-ATTCCAACCATTCTGACTGT-3′ (reverse). GAPDH was 5′-GAAGGTGAAGGTCGGAGTC-3′ (forward) and 5′-GAAGATGGTGATGGGATTTC-3′ (reverse).

### 2.3 Western blot analysis

Treated cells were harvested and extracted using radioimmunoprecipitation assay lysis buffer (50 mM Tris [pH 7.4], 150 mM NaCl, 1% Triton X-100, 1% sodium deoxycholate, 0.1% sodium dodecyl sulfate, and a proteinase inhibitor cocktail). Proteins were separated on 12%–15% SDS-PAGE gel and transferred onto PVDF membranes. The membranes were incubated for 1 h at room temperature with 5% nonfat dried milk in TBST buffer and incubated at 4°C overnight with anti-PRKCI (Sigma-Aldrich, HPA026574, St. Louis, Missouri, USA) anti-SQSTM1 (Cell Signaling Technology, 8025T, Danvers, MA, USA), anti-mTOR (Cell Signaling Technology, 2983T, Danvers, MA, USA), anti-p-mTOR (Ser2448) (Cell Signaling Technology, 2971S, Danvers, MA, USA), anti-AKT (Cell Signaling Technology, 9272S, Danvers, MA, USA), anti-p-AKT (Ser473) (Cell Signaling Technology, 9271T, Danvers, MA, USA), anti-LC3B (Cell Signaling Technology, 3868S, Danvers, MA, USA), and anti-ACTB (Tianjin Sungene, KM9001T, Tianjin, China) antibody. Protein bands were visualized using DyLight 800/DyLight 680-conjugated secondary antibodies (Rockland, 610–145-002/610–144-002/611–145-002/611–144-002, Philadelphia, PA, USA), and infrared fluorescence images were obtained using an Odyssey infrared imaging system (LI-COR Biosciences, NE, USA).

### 2.4 Plasmid construction and transfection

PRKCI complementary DNA (cDNA) was PCR amplified from a cDNA library of U2OS cells using the forward primer P1 (5′-CCGGAATTCATGCCGACCCAGAGGGACA-3′) and reverse primer P2 (5′-CGCGGATCCCGGACACATTCTTCTGCAGACATC-3′) and cloned into a pcDNA3.1 vector. Specific short hairpin RNAs (shRNAs) mediating PRKCI gene knockdown with the targeting sequences 50-CCAGAACACAGAGGAUUAU-30 (PRKCI shRNA1) and 50-GGUGGUACCUCCCUUUAAA-30 (PRKCI shRNA2) were purchased from OriGene Technologies Inc. All plasmids were confirmed by DNA sequencing. Cells were transfected with MegaTran 1.0 Transfection Reagent (OriGene, TT200004, Rockville, MD, USA) according to the manufacturer’s instructions.

### 2.5 Cell proliferation

The Cell Counting Kit-8 (CCK-8) assay was used to evaluate the proliferation of cells. After 24 h (or 48 h) of transfection with the relevant plasmids (as indicated), SW1353 or U2OS cells were seeded into a 96-well plate (3,000 cells/well). A 10-µL aliquot of CCK-8 solution was added to each well and incubated for 2 h. The absorbance at 450 nm was assessed by a microplate reader. Each experiment was performed independently in triplicate.

### 2.6 Colony formation assay

After 24 h (or 48 h) of transfection with the relevant plasmids (as indicated), SW1353 or U2OS cells were seeded into six-well plates (3,000 cells/well) and selected with G418 at 0.3 mg/mL for 20 days. The cells were fixed with 100% methanol for 30 min and dyed with 0.1% crystal violet for 30 min. Cell colonies were counted, and all experiments were performed independently in triplicate.

### 2.7 Cell cycle analysis

We employed the propidium iodide (PI) staining method to analyze the cell cycle. Firstly, cells were collected 48 h after transfection with relevant plasmids, and each group of cells was counted to ensure 1 × 10^6^ cells per group. Subsequently, the cells were washed with PBS and fixed with 70% ethanol. Next, 0.5 mL of PI staining buffer (containing 25 μL of PI dye and 10 μL of RNase A) was added to each cell sample and gently mixed, and the cells were suspended. The cells were incubated in the dark at 37°C for 30 min. The BD Accuri C6 flow cytometer was used to detect the cell cycle distribution, and doublet cells were excluded through pulse processing (which can be achieved by analyzing pulse area and pulse width). The data were then applied to PI histograms. The FlowJo software (Tree Star Inc., Ashland, USA) was utilized to quantify the percentage of cells in each phase of the cell cycle. All experiments were conducted independently, in triplicate.

### 2.8 Cell migration and invasion assay

The Transwell system was used for cell migration and invasion assays. The transfected cells were resuspended in serum-free DMEM and seeded in the upper chamber. In the invasion experiment, the filter was precoated with 100 μL of Matrigel (1:8); however, this process was not carried out in the migration experiment. DMEM containing 15% FBS was added to the lower chamber. After incubation for 24 h, the cells were fixed with methanol for 30 min, stained with 0.1% crystal violet for 30 min, photographed (magnification, ×50), and counted.

### 2.9 Wound-healing assay

The transfected cells were seeded in six-well plates and cultured for 16 h. A 200-μL micropipette tip was used to make a wound with uniform width. The cells were observed at 0 and 24 h after wound formation and photographed with an inverted phase contrast microscope (magnification, ×50). Cell migration was determined by measuring the degree of wound healing.

### 2.10 Human tumor specimens

Tumor specimens were obtained from 62 patients with osteosarcoma treated in the Affiliated Yantai Yuhuangding Hospital of Qingdao University. The study was approved by the Medical Ethics Committee of the Affiliated Hospital of Qingdao University, and patients involved were informed through written informed consent. All these patients underwent surgical treatment, and the tumor tissue and some adjacent nontumor tissues were surgically removed. Tumor specimens were immediately frozen in liquid nitrogen and stored at −80°C in a refrigerator. In addition, the clinical data (such as gender, age, TNM, differentiation, and histological type) of all these patients were collected through electronic medical records.

### 2.11 Immunohistochemistry

Immunohistochemistry analysis was performed as previously described (20). The paraffin sections of the collected samples were analyzed by immunohistochemistry with rabbit anti-PRKCI and anti-SQSTM1 antibodies, respectively. Assigning the percentage of positive tumor cells (0, none; 1, < 20% of positive staining cells; 2, 20%–50% of positive staining cells; 3, > 50% of positive staining cells) scored the extent of PRKCI staining. The evaluation was carried out by two professionals. In cases of disagreement, it should be decided through consultation. PRKCI and SQSTM1 expression scores of 0 and 1 in **Tables 1**, **2** were classified as low expression, and PRKCI and SQSTM1 expression scores of 2 and 3 represented high expression.

**Table 1:** Comparison of PRKCI protein expression in osteosarcoma and cancer-adjacent tissues.

Groups	*N*	PRKCI expression				*p*-value
		0	1	2	3	
Osteosarcoma cancer	42	1	7	6	28	< 0.0001
Cancer adjacent normal bone tissues	42	31	11	0	0	

**Table 2:** Comparison of SQSTM1 protein expression in osteosarcoma cancer and cancer-adjacent tissues.

Groups	*N*	SQSTM1 Expression				*p*-value
		0	1	2	3	
Osteosarcoma cancer	20	1	6	9	4	< 0.0001
Cancer adjacent normal bone tissues	20	12	7	1	0	

### 2.12 Immunoprecipitation

For the immunoprecipitation (IP) analysis, U2OS cells were placed on ice and washed three times with 1 × PBS. Co-IP cell lysate [purchased from Beyotime, P0013J, Beijing, China, added with protease inhibitor cocktail (Roche, 04693116001, Basel, Switzerland)] was added. Cultured cells were scraped, lysed on ice for 30 min, and mixed with the tip of the spear repeatedly at 4°C. The supernatant was centrifuged at 12,000 *g* for 20 min and transferred into a new centrifuge tube. After BCA quantification, 1 μg antibody per 500 μg protein sample was added, precleared, mixed with anti-PRKCI antibody or Control IgG (Cell Signaling Technology, 2729S, Danvers, MA, USA), and incubated overnight (4°C). The cells were then incubated with 25 μL protein G (GE Healthcare, 17-0618-01, Glattbrugg, Switzerland) for an additional 2 h at 4°C and washed five times with wash solution (50 mM Tris, pH 8.0, 150 mM NaCl, 0.4% NP-40 and 5 mM MgCl_2_). Samples were then eluted with 30 μL of 2× SDS loading buffer and denatured at 90°C for 5 min. Immunoblotting was performed using an anti-p62/SQSTM1 (Cell Signaling Technology, 4043, Danvers, MA, USA) antibody.

### 2.13 Statistical analysis

All statistical analyses were performed with GraphPad software. Statistical differences between groups were analyzed using the Student’s *t*-test for continuous variables. Data were reported as the mean ± standard error of the mean (SEM), and *p* < 0.05 was considered statistically significant.

## 3 Results

### 3.1 PRKCI is overexpressed in osteosarcoma cell lines

**Figure 1:**
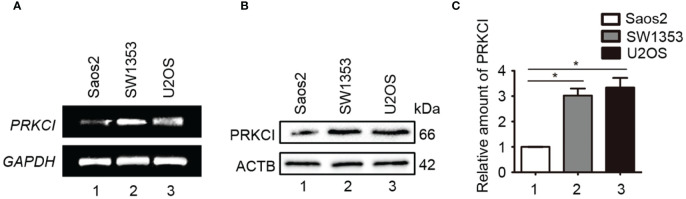
PRKCI was upregulated in osteosarcoma cell lines. **(A, B)** The mRNA and protein expression levels of PRKCI were detected by RT-PCR and Western blot in U2OS, Saos2, and SW1353 cells. **(C)** Quantification of PRKCI protein amounts relative to ACTB in the osteosarcoma cell lines (^*^ *p* < 0.05).

The mRNA and protein expression levels of PRKCI in one osteosarcoma cell line (U2OS), one human osteoblast cell line (Saos2), and one human chondrosarcoma cell line (SW1353) were detected by RT-PCR and Western blot analyses. Results showed that PRKCI was overexpressed obviously in osteosarcoma and chondrosarcoma cell lines compared with osteoblast lines (**Figures 1A–C**), which indicated that PRKCI might play an important role in osteosarcoma tumorigenesis.

### 3.2 Overexpression of PRKCI promotes osteosarcoma cell proliferation *in vitro*

**Figure 2:**
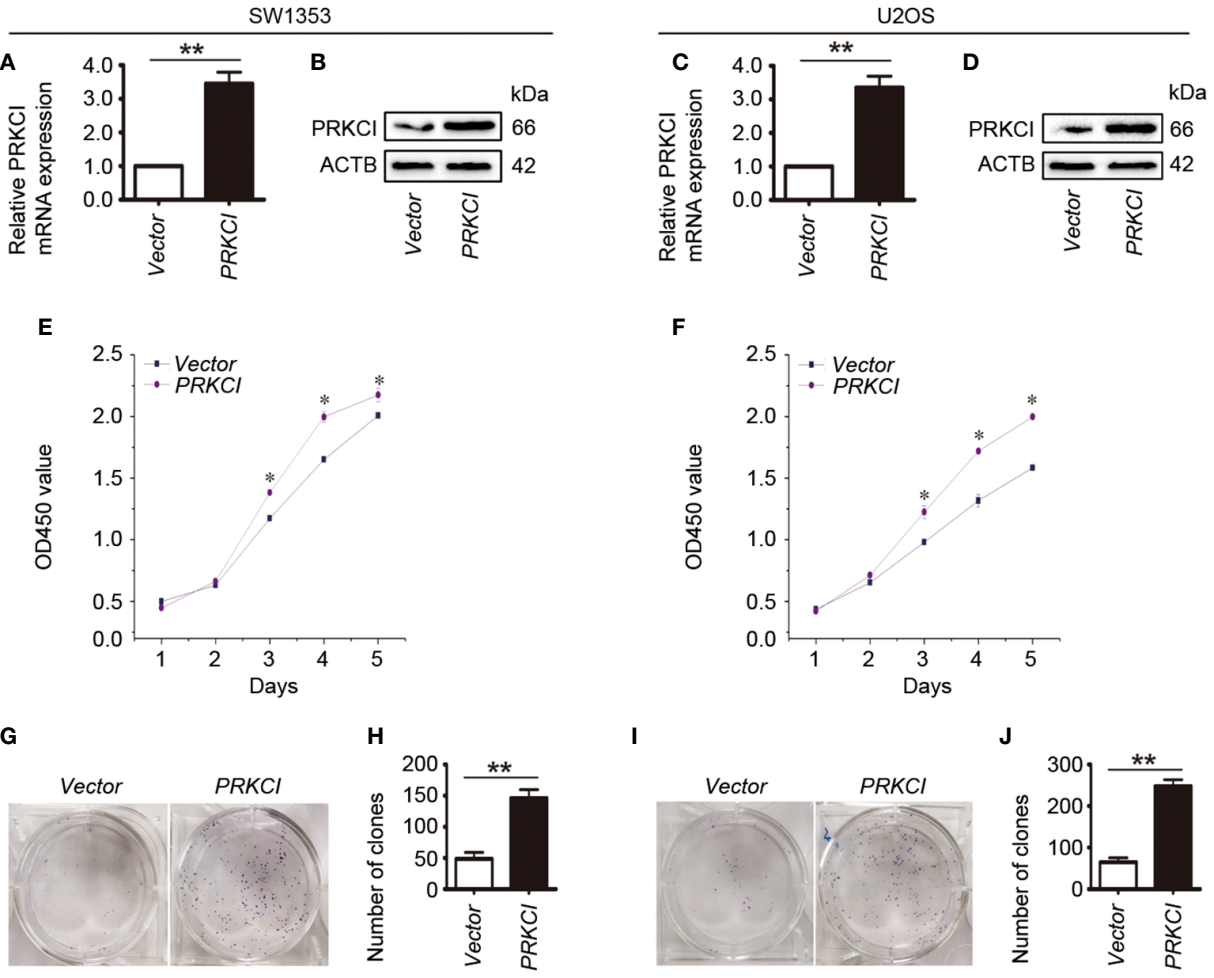
Overexpression of PRKCI promotes osteosarcoma cell growth *in vitro*. **(A–D)** Levels of mRNA and protein expression were validated by RT-PCR and Western blot in SW1353 and U2OS cells transfected with empty vector or PRKCI plasmid for 24 h, respectively. **(E**, **F)** CCK-8 assay was performed in SW1353 and U2OS cells after transfection. **(G–J)** Colony-forming assay was performed in osteosarcoma cells with or without PRKCI overexpression. (Data were representative of results from three independent experiments, ^*^ *p* < 0.05, ^**^ *p* < 0.01).

In order to explore the role of PRKCI in osteosarcoma cells, the effect of PRKCI-overexpression on the proliferation of osteosarcoma cells (SW1353 and U2OS) was studied by the CCK-8 assay and colony-forming assay. **Figures 2A–D** show that PRKCI was overexpressed in osteosarcoma cells after plasmid transfection. **Figures 2E, F** show that osteosarcoma cells transfected with the PRKCI plasmid had stronger proliferation ability than cells transfected with an empty vector (*p* < 0.05), which was time-dependent. From **Figures 2G–J**, we learned that the number of clones in the PRKCI-overexpression group was significantly higher than that in the empty vector group (*p* < 0.01), indicating that the overexpression of PRKCI significantly increased the colony-forming capacity of cells (SW1353 and U2OS).

### 3.3 Knockdown of PRKCI inhibits osteosarcoma cell proliferation *in vitro*

In order to further clarify the effect of PRKCI on the biological characteristics of osteosarcoma cells, the changes in SW1353 and U2OS cell proliferation after silencing PRKCI were investigated. We identified two effective shRNAs against PRKCI (PRKCI shRNA1 and PRKCI shRNA2) that had similar effects in a previous paper published by our lab (21). Here, we used PRKCI shRNA2 as the representative shRNA to perform the subsequent experiments. RT-PCR and Western blot assays were used to verify the gene knockdown of PRKCI.

**Figure 3:**
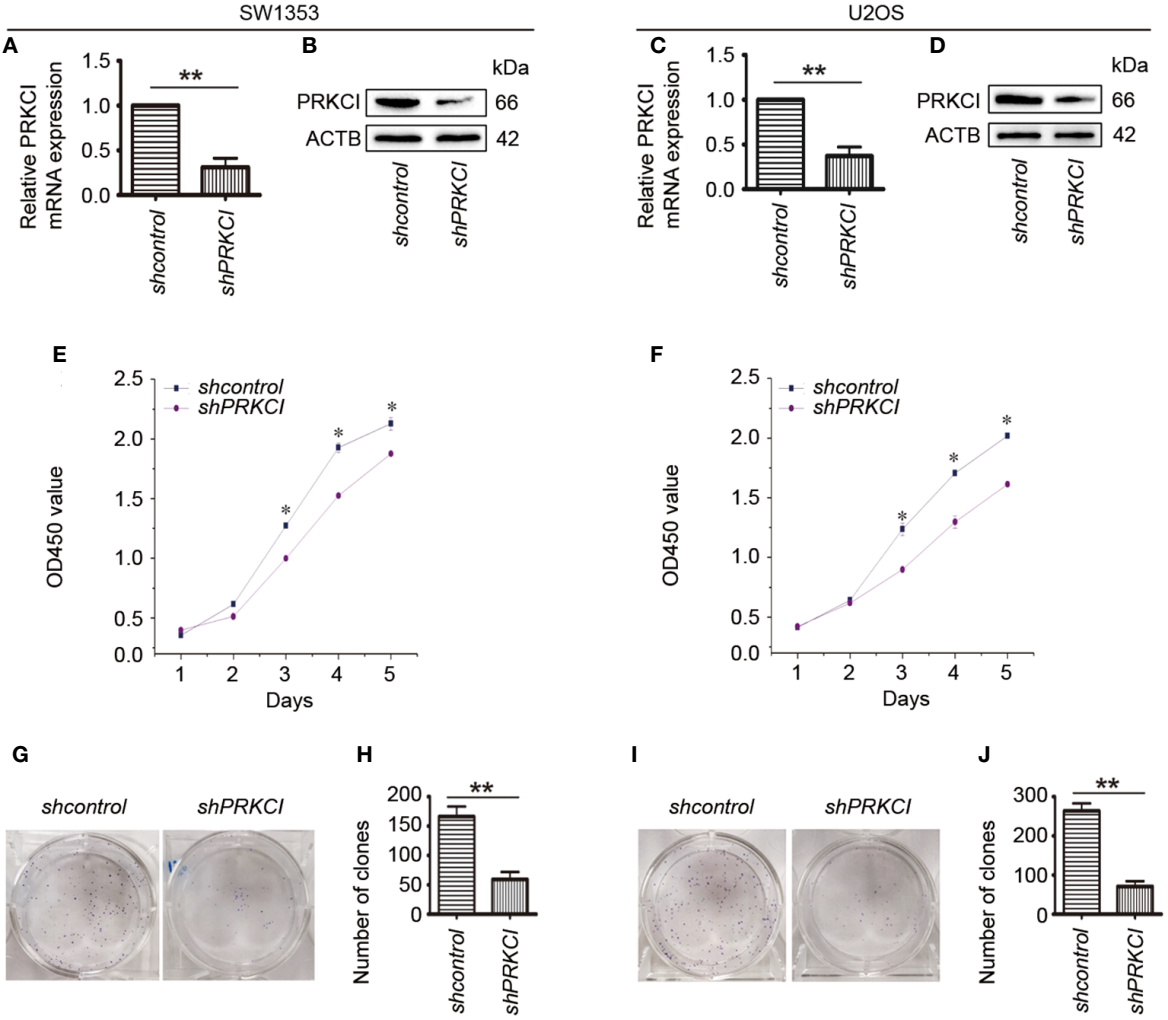
Knockdown of PRKCI inhibited osteosarcoma cell growth *in vitro*. **(A–D)** Levels of mRNA and protein expression of PRKCI were validated by RT-PCR and Western blot in SW1353 and U2OS cells transfected with shcontrol or shPRKCI for 48 h. **(E**, **F)** CCK-8 assay was performed after shRNA transfection. **(G–J)** Colony-forming assay was performed in osteosarcoma cells with or without PRKCI-silenced. (Data were representative of results from three independent experiments, ^*^ *p* < 0.05, ^**^ *p* < 0.01).

The mRNA and protein expression levels of PRKCI were significantly decreased after transfection with PRKCI-specific shRNA in SW1353 and U2OS cells (**Figures 3A–D**). Subsequently, the CCK-8 assay showed that knockdown of PRKCI significantly inhibited the growth of osteosarcoma cells with time dependence (**Figures 3E, F**). Results of colony-forming assay revealed that the number and size of colonies were both obviously decreased in the PRKCI-knockdown group compared with the control group (**Figures 3G–J**). These results made clear that PRKCI played an oncogenic role in osteosarcoma.

### 3.4 Knockdown of PRKCI arrests cell cycle at G2/M phase in osteosarcoma cells

**Figure 4:**
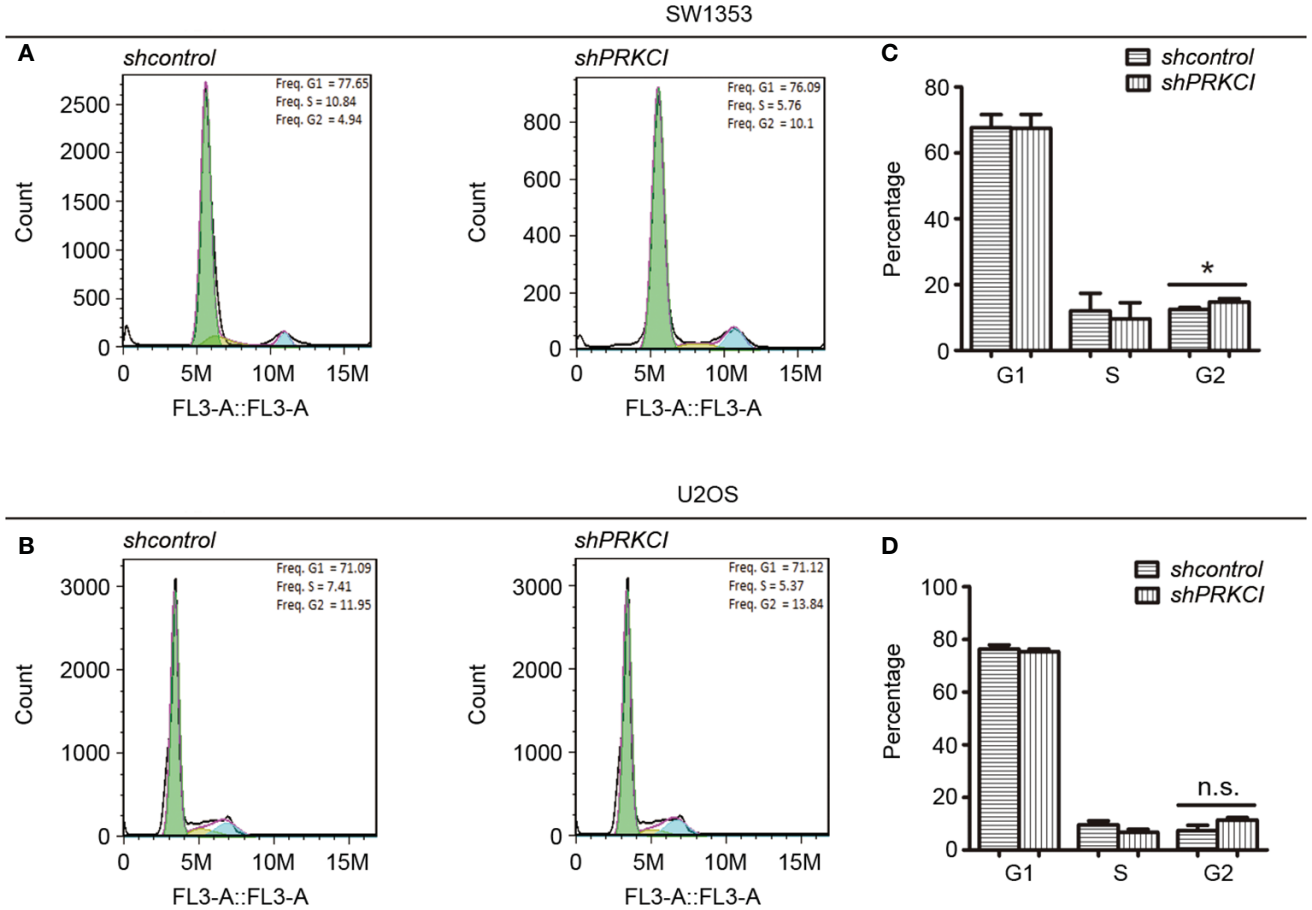
Knockdown of PRKCI-arrested cell cycle at G2/M phase in osteosarcoma cells. The cell cycle results of SW1353 **(A)** and U2OS **(B)** cells transfected with shcontrol or shPRKCI for 48 h were analyzed by flow cytometry. **(C, D)** Diagrams showing the results of the cell cycle assays for SW1353 and U2OS cells treated as in **(A)**. ^*^ *p* < 0.05, n.s., no significant differences.

Flow cytometry was used to determine the changes in the cell cycle in osteosarcoma cells after the knockdown of PRKCI. Results revealed that shPRKCI-transfected cells had a significantly higher percentage of cells in the G2/M phase than that of shcontrol-transfected cells in SW1353 (**Figures 4A, B**). Meanwhile, there was no significant difference in the G1 or S cell populations between PRKCI-knockdown and control-transfected cells. In U2OS cells, silencing PRKCI did not significantly induce G2/M cell cycle arrest, with no significant statistical difference (**Figures 4C, D**). This suggested that the knockdown of PRKCI inhibited the proliferation of osteosarcoma cells by arresting the cell cycle at the G2/M phase in SW1353 cells, with barely a difference in U2OS cells.

### 3.5 Knockdown of PRKCI inhibits osteosarcoma cell migration and invasion

**Figure 5:**
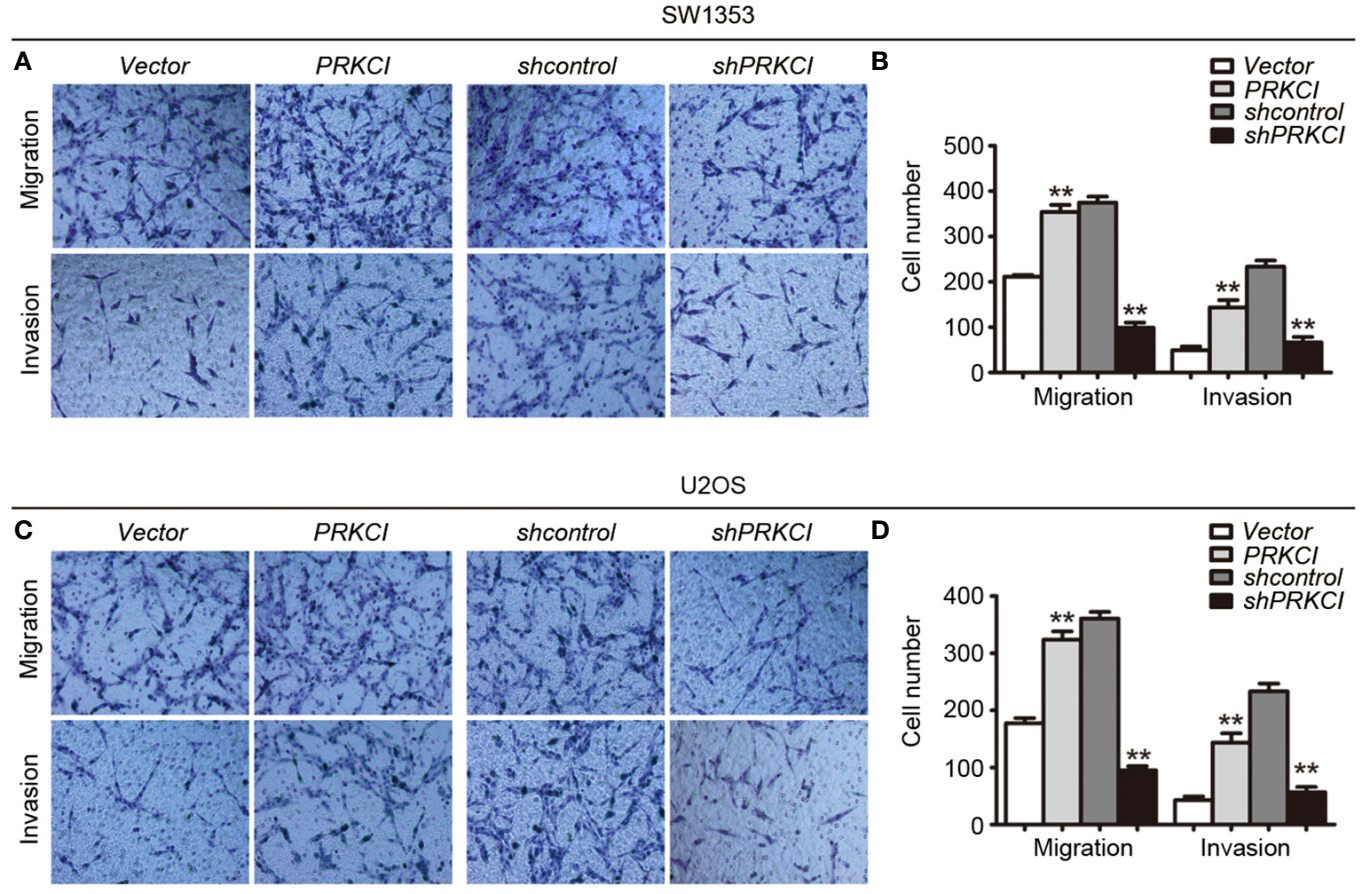
The Transwell system was used to evaluate the effect of PRKCI on the migration and invasion of osteosarcoma cells. **(A**, **C)** Transwell migration assay and Matrigel invasion assay for SW1353 cells or U2OS cells after transfection empty vector or PRKCI plasmid for 24 h (shcontrol or shPRKCI for 48 h). Cells were stained with crystal violet (magnification: ×200). **(B**, **D)** Quantification of invaded and migrated SW1353 cells or U2OS cells. (Data were based on three independent experiments and shown as the mean ± SEM, ^**^ *p* < 0.01).

**Figure 6:**
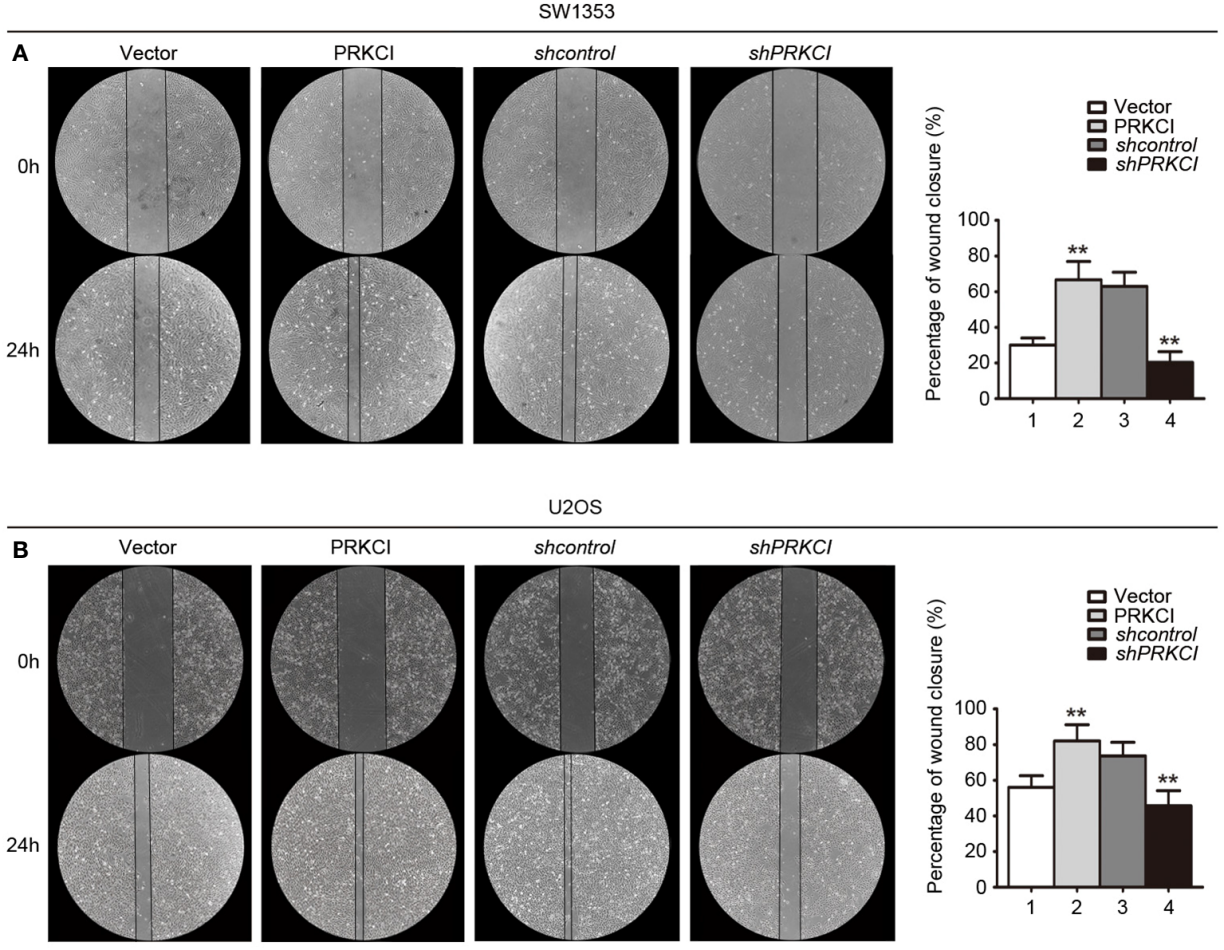
A wound-healing assay was used to evaluate the effect of PRKCI on the migration of osteosarcoma cells. **(A, B)** Microscopic images of wound-healing assay data for SW1353 and U2OS cells transfected with empty vector or PRKCI plasmid for 24 h (shcontrol or shPRKCI for 48 h). (Data were based on three independent experiments and shown as the mean ± SEM, **p < 0.01).

The migration and invasion of osteosarcoma cells were first detected by the Transwell system in the presence of PRKCI-overexpression and PRKCI-silencing. **Figure 5** shows that, compared with control groups, overexpression of PRKCI could promote the migration and invasion of osteosarcoma cell lines SW1353 and U2OS, while silencing of PRKCI reversed this effect (*p* < 0.01). A wound-healing assay is another commonly used method to evaluate cell migration. The results in **Figure 6** revealed that PRKCI overexpression and PRKCI silencing showed the same effect as before in both cell lines. These results suggested that PRKCI might be involved in the growth and metastasis of osteosarcoma.

### 3.6 PRKCI is highly expressed in osteosarcoma, and the expression is related to histological types in osteosarcoma

**Figure 7:**
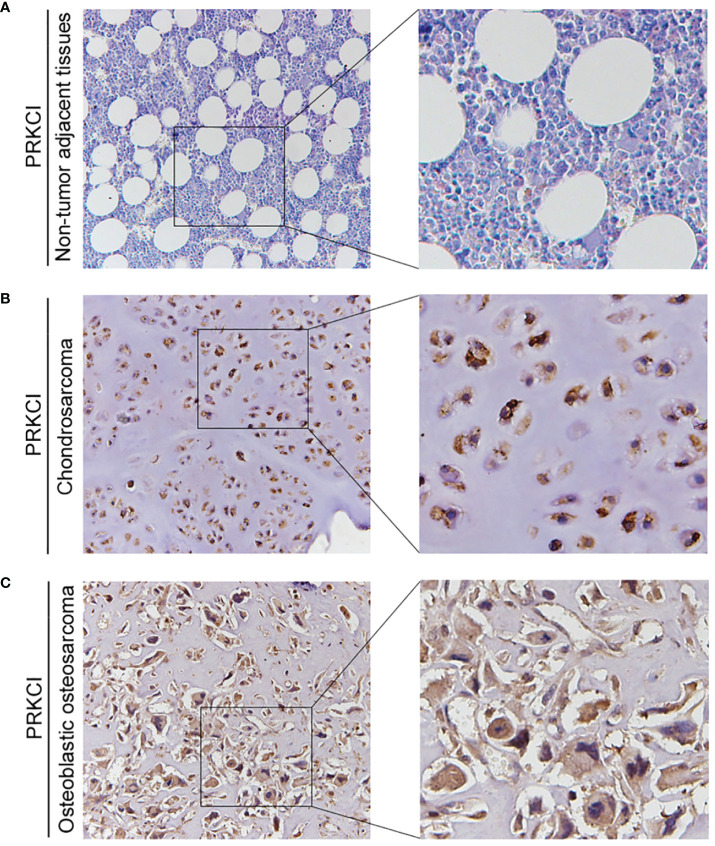
The expression of PRKCI in patients with osteosarcoma. **(A–C)** Representative IHC image of PRKCI expression in nontumor adjacent tissues, chondrosarcoma tissues, and osteoblastic osteosarcoma tissues. (Original magnification: ×50 and ×200).

Based on the biological role of PRKCI in osteosarcoma cell lines, the expression of PRKCI in osteosarcoma tissues and adjacent tissues of 42 patients was detected by the IHC to further explore its role in human osteosarcoma. The grade of samples was divided according to the ratio of PRKCI-positive cell rate in tissues. By comparison, we found that the expression of PRKCI in osteosarcoma was much higher than that in corresponding nontumor tissues (*p* < 0.0001) (**Table 1**). Furthermore, the correlation between PRKCI expression and the clinical characteristics of osteosarcoma patients was explored. **Table 3** shows that the expression level of PRKCI was significantly correlated with histological types (*p* = 0.0015), but not with other demographic and clinical factors (including gender, age, TNM, and differentiation). The expression of PRKCI in osteosarcoma patients is shown in **Figure 7**, from which we could intuitively see that compared with nontumor adjacent tissues, the expression of PRKCI in osteosarcoma was significantly upregulated, and the expression in osteoblastic osteosarcoma was higher than that in chondrosarcoma. Taken together, these results indicated that PRKCI played an important role in osteosarcoma and was related to the malignancy of osteosarcoma.

**Table 3:** The correlation between PRKCI expression and clinical characteristics of osteosarcoma patients.

Variables	Cases	PRKCI lower expression (*n* = 8)		PRKCI higher expression (*n* = 34)		*p*-value
		*N*	%	*N*	%	
Sex						
Male	23	6	26.0	17	74.0	0.3933
Female	19	2	10.5	17	89.5	
Age (years)						
< 50 years	36	7	19.4	29	80.6	0.8858
≥50 years	6	1	16.7	5	83.3	
TNM						
T1	14	4	28.6	10	71.4	0.3817
T2	28	9	32.1	19	67.9	
Differentiation						
Well	20	4	20.0	16	80.0	0.7371
Poor/Moderate	22	4	18.2	18	81.2	
Histological types						
Osteoblastoma	13	5	38.5	8	61.5	0.0007^***^
Chondroblastoma	20	0	0.0	20	100.0	
Others	9	3	33.3	6	66.7	

^*^p < 0.05; ^**^p < 0.01; ^***^p < 0.001.

### 3.7 Silencing PRKCI inhibits the proliferation of osteosarcoma cells via the Akt-mTOR signaling pathway, and the phosphorylation level of mTOR is increased in osteosarcoma patients

**Figure 8:**
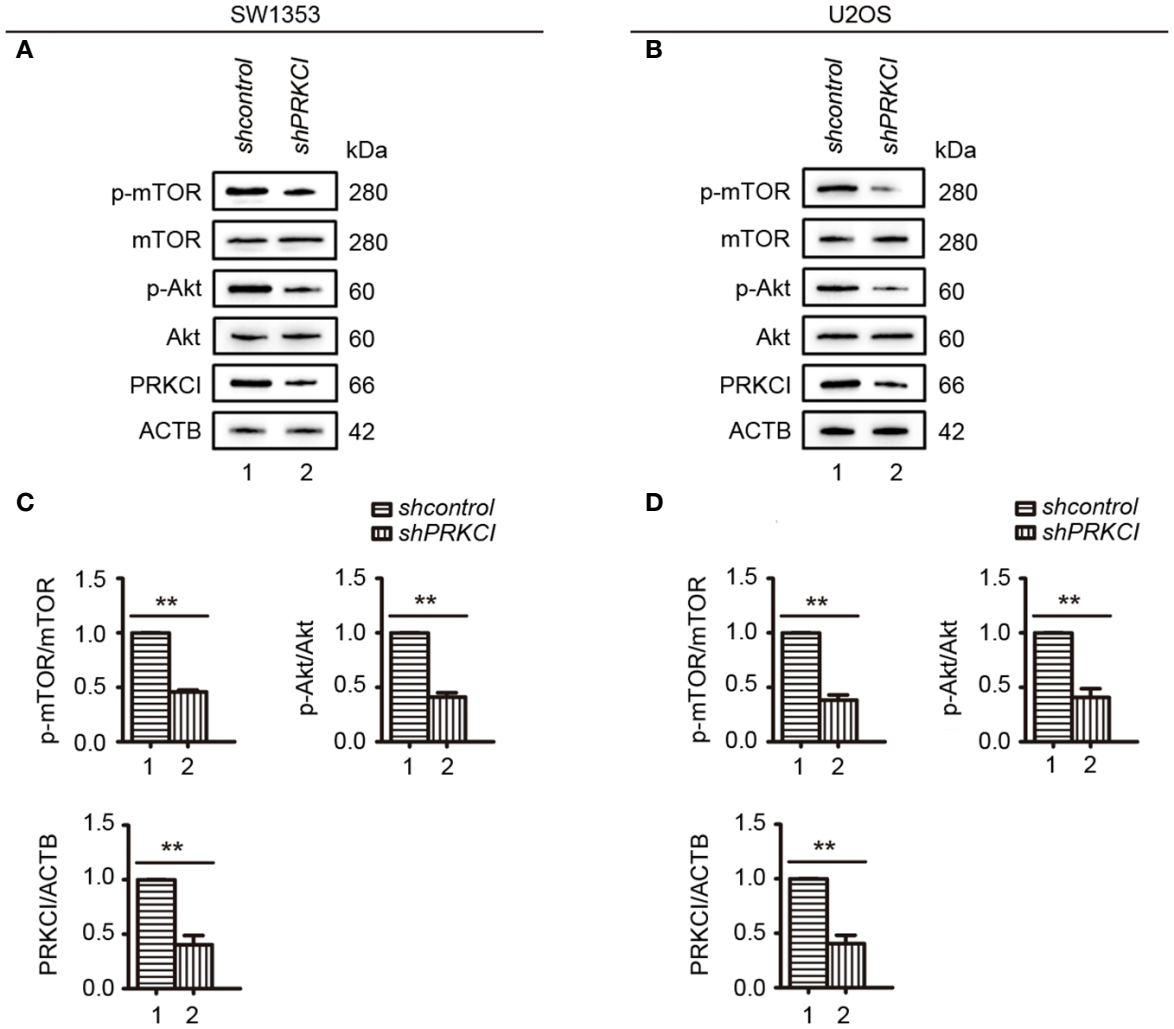
Knockdown of PRKCI inhibited the Akt-mTOR signaling pathway in osteosarcoma cells. **(A, B)** Western blot analysis of the Akt-mTOR signaling pathway, including total and phosphorylation levels of mTOR (Ser2448) and Akt (Ser473), in SW1353 and U2OS cells transfected with shcontrol or shPRKCI for 48 h **(C, D)** Quantification of the relative amounts of phosphorylated mTOR (p-mTOR) to total mTOR, phosphorylated AKT (p-AKT) to total AKT, and PRKCI to ACTB (β-actin) in SW1353 and U2OS cells transfected with indicated plasmids. Data are shown as the mean ± SEM of at least three independent experiments ^(**^ *p* < 0.01).

Some studies have pointed out that the Akt-mTOR signaling pathway, downstream of PRKCI, promotes the malignant progression of laryngeal squamous cell carcinoma and inhibits autophagy (21, 22). Here, to determine whether they are pathway molecules for PRKCI to inhibit the proliferation of osteosarcoma cells, we detected the molecule expression of Akt-mTOR in SW1353 and U2OS cells when PRKCI was knocked down. Results showed that mTOR and Akt phosphorylation were significantly reduced in PRKCI-silenced cells (**Figures 8A–D**).

**Figure 9:**
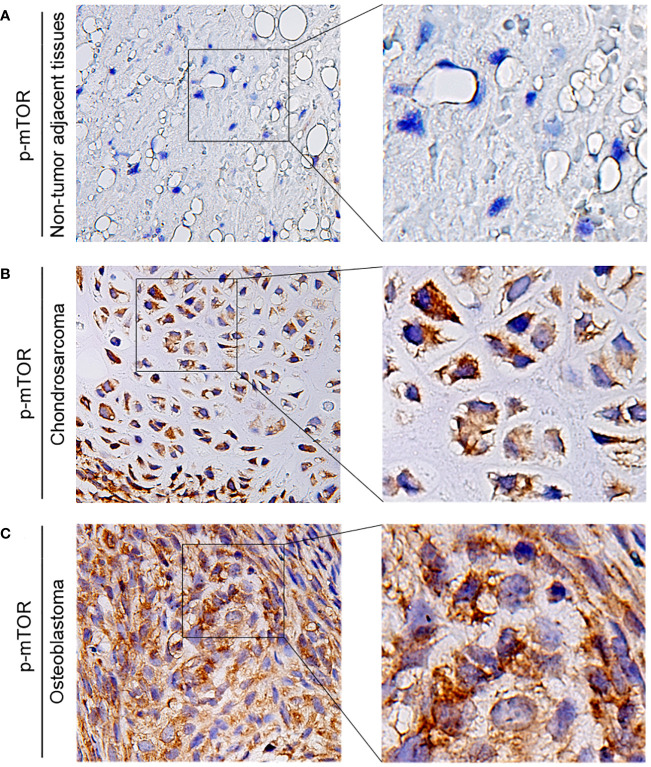
The expression of p-mTOR in patients with osteosarcoma. **(A–C)** Representative IHC image of p-mTOR expression in nontumor adjacent tissues, chondrosarcoma tissues, and osteoblastic osteosarcoma tissues. Original magnification: ×50 and ×200.

The expression of p-mTOR in osteosarcoma tissues and adjacent normal tissues of 20 patients was detected by the IHC to further explore its role in human osteosarcoma. We found that the phosphorylation of mTOR was increased in osteosarcoma patients (**Figures 9A–C**).

Overall, these results suggest that PRKCI depletion inhibits osteosarcoma cell growth via inactivation of the Akt-mTOR pathway. Moreover, the phosphorylation level of mTOR in osteosarcoma patients increased.

### 3.8 SQSTM1 is highly expressed in osteosarcoma and interacts with PRKCI

**Figure 10:**
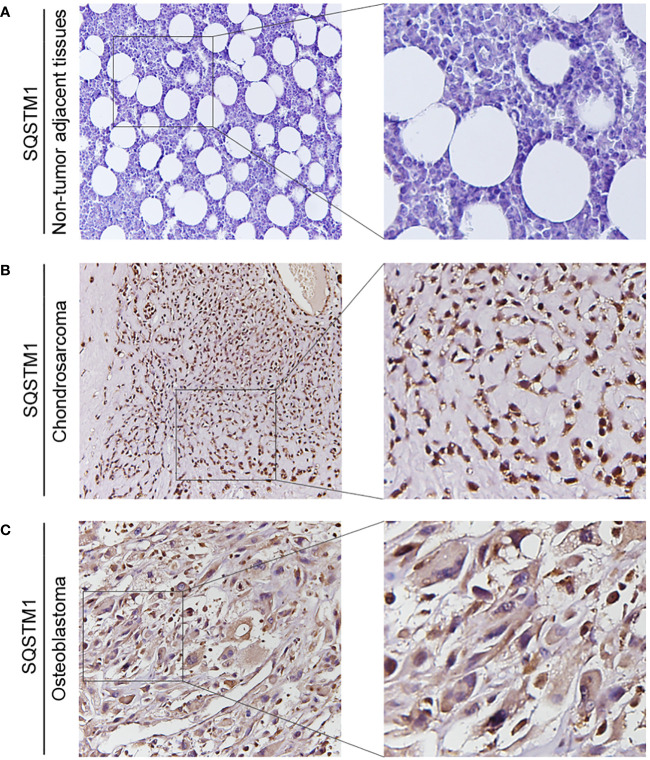
The expression of SQSTM1 in patients with osteosarcoma. **(A–C)** Representative IHC image of SQSTM1 expression in nontumor adjacent tissues, chondrosarcoma tissues, and osteoblastic osteosarcoma tissues. Original magnification: ×50 and ×200.

The expression of SQSTM1 in osteosarcoma tissues and adjacent tissues of 20 patients was detected by the IHC to further explore its role in human osteosarcoma. The samples were graded based on the proportion of SQSTM1-positive cells present in tissues. Upon comparison, it was observed that the expression of SQSTM1 was significantly higher in osteosarcoma tissues than in the corresponding nontumor tissues (*p* < 0.0001), as shown in **Table 2**. In addition, the study examined the association between the expression level of SQSTM1 and the clinicopathological characteristics of osteosarcoma. **Table 4** indicates no significant correlation between SQSTM1 expression and demographic or clinical factors, including gender, age, TNM, differentiation, and histological types. **Figure 10** reveals the SQSTM1 expression in osteosarcoma patients. The comparison between nontumor adjacent tissues and osteosarcoma strongly suggested a significant upregulation of SQSTM1 expression in the latter. Moreover, osteoblastic osteosarcoma exhibited a higher expression of SQSTM1 compared to chondrosarcoma.

**Table 4:** Relationship between SQSTM1 expression level and clinicopathological features in osteosarcoma (*n* = 20).

Variables	Cases	SQSTM1 lower expression (*n* = 7)		SQSTM1 higher expression (*n* = 13)		*p*-value
		*N*	%	*N*	%	
Sex						
Male	18	6	33.3	12	66.7	0.7309
Female	2	1	50.0	1	50.0	
Age (years)						
< 50 years	16	5	31.3	11	68.7	0.8975
≥ 50 years	4	2	50.0	2	50.0	
Tumor						
T1	9	4	44.4	5	55.6	0.5322
T2	11	3	27.3	8	72.7	
Differentiation						
Well	10	4	40.0	6	60.0	0.2954
Poor/Moderate	10	3	30.0	7	70.0	
Histological types						
Osteoblastic	6	2	33.3	4	66.7	0.7871
Chondroblastic	8	3	37.5	5	62.5	
Others	6	2	33.3	4	66.7	
TNM stage						
I	5	1	20.0	4	80.0	1.0000
II	15	6	40.0	9	60.0	

**Figure 11:**
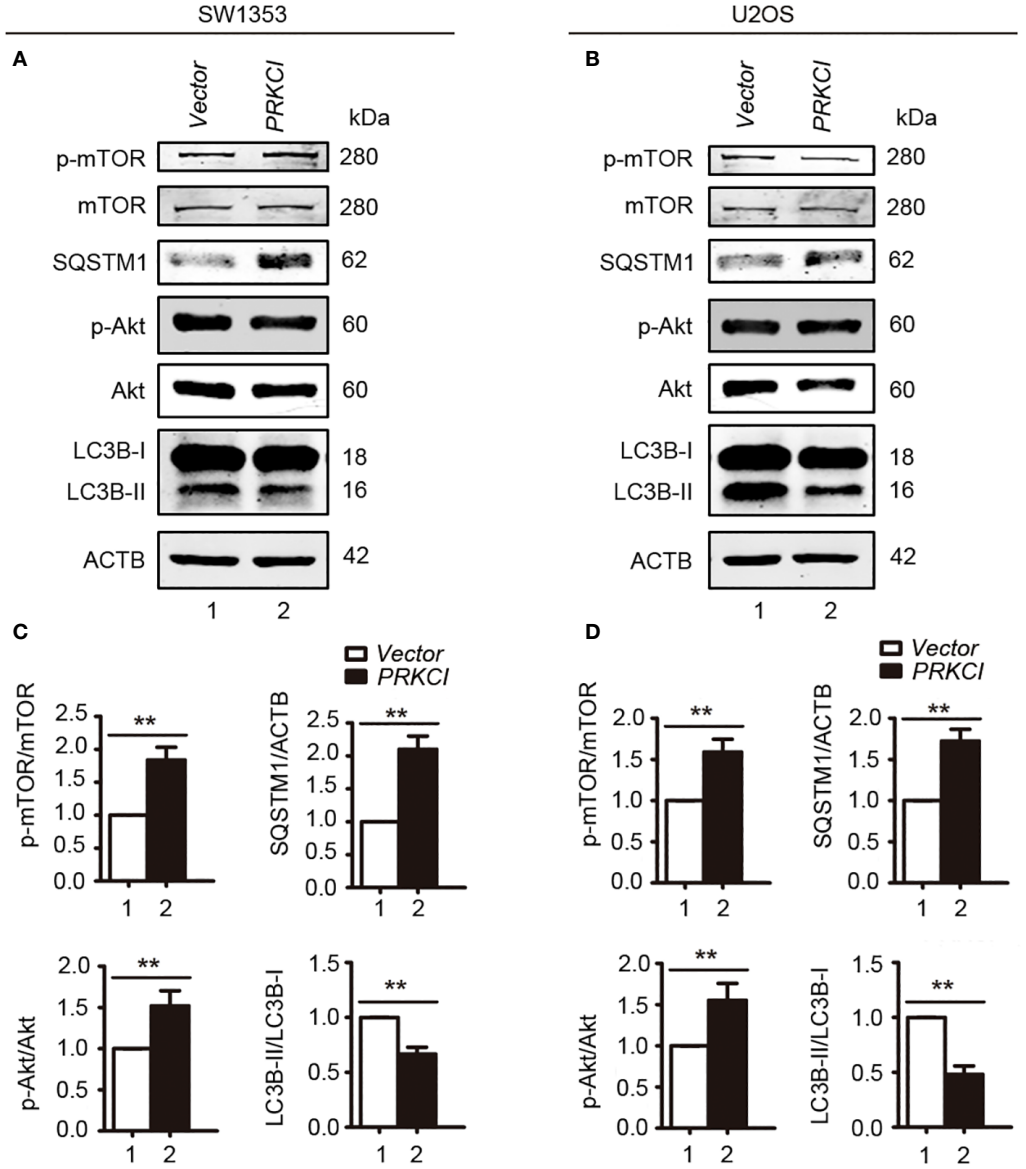
Overexpression of PRKCI enhanced the SQSTM1 and Akt-mTOR signaling pathways and inhibited the level of autophagy in osteosarcoma cells. **(A, B)** Western blot analysis of the Akt-mTOR signaling pathway, including total and phosphorylation levels of mTOR (Ser2448), Akt (Ser473), SQSTM1, and LC3B-I/II in SW1353 and U2OS cells transfected with vector or PRKCI for 24 (h) **(C, D)** Quantification of the relative amounts of p-mTOR to total mTOR, SQSTM1 to ACTB, p-AKT to total AKT, and LC3B-II to LC3B-I in SW1353 and U2OS cells transfected with indicated plasmids. Data are shown as the mean ± SEM of at least three independent experiments (^**^ *p* < 0.01).

As shown in **Figure 11**, overexpression of PRKCI enhanced the level of SQSTM1, p-mTOR, and p-AKT and inhibited the level of LC3B-II, indicating that overexpression of PRKCI could enhance the level of SQSTM1 and inhibit the level of autophagy through the Akt-mTOR pathway in osteosarcoma cells.

**Figure 12:**
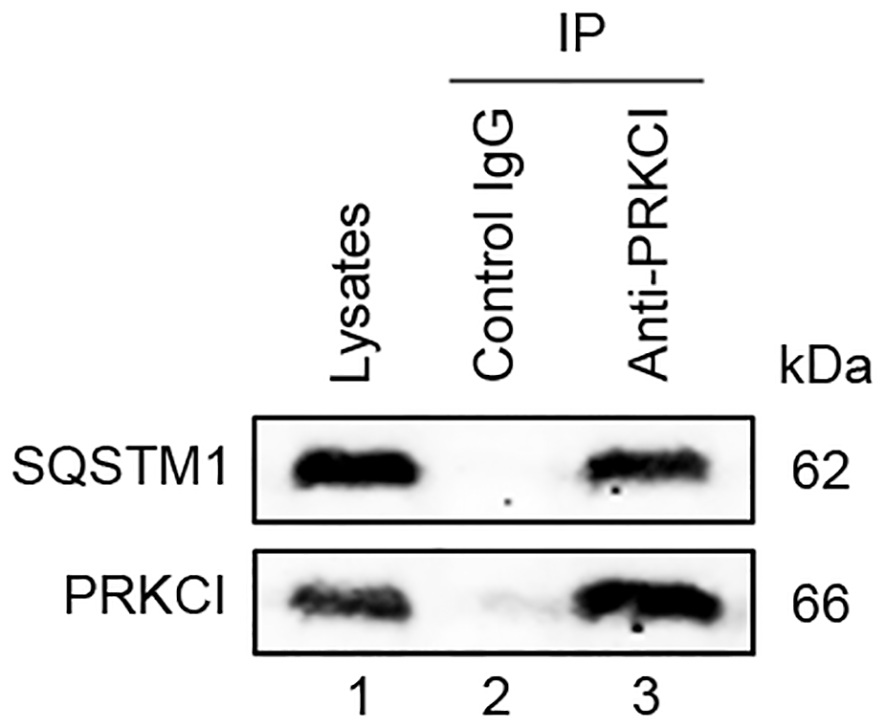
PRKCI interacted with SQSTM1. The whole cell lysate of U2OS was immunoprecipitated with anti-PRKCI and control IgG, and the expression of SQSTM1 protein in the immunoprecipitates was detected by Western blot.

The interaction between SQSTM1 and PRKCI in U2OS cells is shown in **Figure 12**. These findings indicated the crucial role of SQSTM1 in osteosarcoma.

## 4 Discussion

Osteosarcoma is a common malignant tumor in children and adolescents, which contributes greatly to the mortality of childhood cancer (23). Its incidence is the highest in adolescence, reaching 8–11 per million in adolescents aged 15–19 years, and it accounts for 15% of all extracranial cancers in this age group (24). Surgery and chemotherapy have greatly improved the survival rate of osteosarcoma to nearly 70%, but its survival rate has not changed in recent decades (3, 25). Osteosarcoma has high genomic instability, genetic mutations, and interference of multiple signaling pathways (26). Many factors, such as bone development, tumor microenvironment, gene variation, and signaling pathway have been identified as contributing to the occurrence and development of osteosarcoma (27). Targeting the molecular mechanism of osteosarcoma is one of the important strategies for future treatment. Trastuzumab, a Her2 monoclonal antibody, has been used in the treatment of breast cancer. Studies have shown that high expression of the Her2 protein in osteosarcoma patients and cell lines suggests that trastuzumab may be effective in osteosarcoma (28). The role of EGFR (HER1) in osteosarcoma has also attracted attention (29). To sum up, searching for the potential molecular mechanism of osteosarcoma is very important for targeted therapy. In this study, we explored the tumorigenic effect of PRKCI in osteosarcoma by cell experiment and histological analysis.

PKCι (PRKCI) is one of the members of the atypical protein kinase C (aPKC) subfamily, and it is required for embryonic development (30). Some studies have also pointed out that the deficiency of PRKCI in the liver will increase insulin sensitivity while deficiency in β-cell will damage glucose tolerance (31). Moreover, the biological function of PRKCI in tumors has been widely studied. It is believed that PRKCI can be used as a therapeutic target for ovarian cancer based on gene overexpression and immunosuppression in ovarian cancer (9, 10). PKCι was considered to be associated with a poor prognosis in lung adenocarcinoma, while another atypical protein kinase enzyme, PKC ζ, did not show significant clinical impact (32). Other studies believe that PRKCI is an oncogene of lung cancer and is essential to maintaining the transformed phenotype of human nonsmall cell lung cancer cells (33). However, the role of PRKCI in osteosarcoma has not been reported. He et al. found that downregulating of PKC family member PKC-δ could inhibit the development of osteosarcoma (34), while Ren et al. reported the contribution of other members of PKC family (not PRKCI) to lung metastasis of osteosarcoma (35). In short, the effect of PRKCI on the tumorigenicity of osteosarcoma has not been studied. In our study, we found that PRKCI was overexpressed in osteosarcoma cell lines and verified the abnormal expression of PRKCI in osteosarcoma tissues compared with nontumor tissues. Furthermore, overexpression of PRKCI promoted the proliferation and colony-forming capacity of osteosarcoma cells while silencing PRKCI inhibited the proliferation, colony-forming capacity, migration, and invasion of osteosarcoma cells. At the same time, the expression of PRKCI in tumor tissue was only related to histological type, indicating that PRKCI was related to the tumorigenicity of osteosarcoma.

SQSTM1 acts as a receptor for selective autophagy, shuttling ubiquitinated cargoes for degradation (36). It also plays a significant role in tumors. It has been found to be involved in DNA damage and immunotherapy responses in solid tumors (37). Moreover, PRKCI overexpression leads to an increase in the accumulation of the autophagy substrate SQSTM1 in cells, indicating a decrease in the clearance of SQSTM1 (21). PRKCI exerts a negative regulatory effect on the process of autophagy by means of the creation of a ternary complex involving PRKCI, SQSTM1, and LC3 (13). The interaction among PRKCI and SQSTM1 coordinates the oxidative metabolic reaction in hepatic neoplasm (13). However, the interaction between PRKCI and SQSTM1 in osteosarcoma remains unclear. In our investigation, we confirmed the abnormal expression of SQSTM1 in osteosarcoma tissues compared with nontumor tissues. Simultaneously, the expression of SQSTM1 in tumor tissues was not related to clinical characteristics, and SQSTM1 interacted with PRKCI in U2OS cells, suggesting that SQSTM1 and PRKCI played a crucial role in osteosarcoma.

The Akt/mTOR signaling pathway is abnormally expressed in a variety of cancers and participates in various biological characteristics of cancer cells, such as proliferation, apoptosis, metastasis, autophagy, etc. Zheng et al. uncovered a novel mechanism in which TRIM26 impedes the progression of clear cell renal cell carcinoma (ccRCC) by binding to and destabilizing ETK, which inhibits the proliferation, migration, invasion, and epithelial-to-mesenchymal transition (EMT) of ccRCC cells, ultimately resulting in the deactivation of the AKT/mTOR signaling pathway. TRIM26 emerges as a promising candidate for both therapeutic targeting and as a prognostic biomarker for patients suffering from ccRCC (38). Fang et al. discovered that NTNG1 directly interacts with GAS6/AXL to activate the Akt pathway, upregulate RAD51 expression, and strengthen the repair mechanisms of double-strand breaks, ultimately leading to the development of cisplatin resistance (39).

The role of the Akt/mTOR signaling pathway in osteosarcoma has been extensively investigated, revealing that the augmentation of this signaling cascade holds paramount significance in inhibiting autophagy and accelerating proliferation, invasion, metastasis, cell cycle progression, and the overall progression of osteosarcoma (40–42).

Cheng et al. indicated that the knockdown of BZW2 and EEF1D could inhibit the proliferation of osteosarcoma through the Akt/mTOR signaling pathway (19, 43). Sun et al. found that the overexpression of YME1L stimulated osteosarcoma cell growth, likely by sustaining mitochondrial function and activating the Akt-mTOR pathway (40). Zhu et al. demonstrated that XL388, a dual mTORC1/2 inhibitor, displayed potent cytotoxic, cytostatic, and apoptotic effects in various osteosarcoma cell lines. Furthermore, its capacity to block Akt-mTOR activation significantly hindered the growth of osteosarcoma xenografts in nude mice, indicating the potential therapeutic value of concurrent mTORC1/2 inhibition with XL388 in osteosarcoma treatment (44).

Emerging studies have underscored the pivotal importance of PRKCI in triggering Akt-mTOR activation within cancerous cells, revealing its potential as a crucial factor in cancer development and progression. Wang et al. showed that EIF4A3-mediated circPRKCI facilitated cancer progression by modulating WBP2 and activating the AKT signaling pathway, suggesting a potential novel therapeutic strategy for triple-negative breast cancer (45). Abdelatty et al. demonstrated that PKCι triggers the apoptosis and progress of pancreatic cancer cells by activating AKT and Wnt/β-catenin signaling pathways, which provides a potential therapeutic target for the treatment of pancreatic cancer (46). Gao et al. revealed that circPARD3, a unique autophagy inhibitor, promotes the progression and chemoresistance of laryngeal squamous cell carcinoma via the PRKCI-Akt-mTOR axis. This discovery offers fresh perspectives on circRNA-driven autophagy modulation, suggesting circPARD3 as a potential biomarker and therapeutic candidate for laryngeal squamous cell carcinoma (22).

Here, we have found that the Akt-mTOR signaling pathway was involved in the inhibitory effect of PRKCI on osteosarcoma *in vitro*. Despite the fact that surgical resection combined with adjuvant therapies, including chemotherapy, can significantly improve the prognosis of primary osteosarcoma, which achieves a 5-year survival rate of approximately 70%, the overall survival rate for recurrent or metastatic osteosarcoma drops dramatically to around 25% due to factors such as chemotherapy resistance and surgical limitations (47). Our current research elucidated the interaction between PRKCI and SQSTM1, demonstrating that PRKCI upregulated SQSTM1 and mediated critical functions in the proliferation and progression of osteosarcoma cells through the activation of the Akt/mTOR signaling pathway. This laid the foundation for exploring innovative therapeutic strategies targeting PRKCI for the treatment of osteosarcoma. Our research offered promising prospects for developing inhibitors or exploring alternative therapies for future treatments to mitigate osteosarcoma progression and enhance osteosarcoma therapy.

## 5 Conclusions

In conclusion, our study found that PRKCI was overexpressed in osteosarcoma cell lines and tissues and promoted the proliferation and migration of cancer cells. SQSTM1, which interacted with PRKCI, was also overexpressed in osteosarcoma. It suggested that PRKCI was closely related to the tumorigenicity of osteosarcoma, and the Akt/mTOR signaling pathway was involved in this process.

## Data availability statement

The datasets presented in this study can be found in online repositories. The names of the repository/repositories and accession number(s) can be found in the article/supplementary material.

## Ethics statement

The studies involving humans were approved by Medical Ethics Committee, Affiliated Hospital of Qingdao University. The studies were conducted in accordance with the local legislation and institutional requirements. The participants provided their written informed consent to participate in this study.

## Author contributions

LQ: Investigation; Writing – original draft. YX: Investigation; Writing – original draft. JF: Formal analysis; Writing – original draft. XR: Formal analysis; Writing – original draft. ZL: Formal analysis; Writing – original draft. XC: Formal analysis; Writing – original draft. GM: Formal analysis; Writing – original draft. JC: Conceptualization; Supervision; Funding acquisition; Writing – review & editing. CS: Conceptualization; Supervision; Funding acquisition; Writing – review & editing. YL: Conceptualization; Supervision; Funding acquisition; Writing – review & editing.

## References

[1] Ottaviani G Jaffe N. The epidemiology of osteosarcoma. Cancer Treat Res. (2009) 152:3–13. doi: 10.1007/978-1-4419-0284-9_1 20213383

[2] Yu H Qu G Wang Y Mai W Bao JJ Song C. The expression of Eps15 homology domain 1 is negatively correlated with disease-free survival and overall survival of osteosarcoma patients. J Orthop Surg Res. (2019) 14:103. doi: 10.1186/s13018-019-1137-6 30975166 PMC6460645

[3] Pathak AB Advani SH Iyer RS Pai SK Gopal R Nadkarni KS. Adjuvant chemotherapy for osteogenic sarcoma of the extremity with sequential adriamycin and cisplatin. J Surg Oncol. (1993) 52:181–4. doi: 10.1002/jso.2930520313 8441277

[4] Link MP Goorin AM Miser AW Green AA Pratt CB Belasco JB. The effect of adjuvant chemotherapy on relapse-free survival in patients with osteosarcoma of the extremity. N Engl J Med. (1986) 314:1600–6. doi: 10.1056/NEJM198606193142502 3520317

[5] Glover J Krailo M Tello T Marina N Janeway K Barkauskas D. A summary of the osteosarcoma banking efforts: a report from the Children’s Oncology Group and the QuadW Foundation. Pediatr Blood Cancer. (2015) 62:450–5. doi: 10.1002/pbc.25346 PMC4304398 25611047

[6] Ma H Seebacher NA Hornicek FJ Duan Z. Cyclin-dependent kinase 9 (CDK9) is a novel prognostic marker and therapeutic target in osteosarcoma. EBioMedicine. (2019) 39:182–93. doi: 10.1016/j.ebiom.2018.12.022 PMC6355967 30579871

[7] Zhao Z Jin G Yao K Liu K Liu F Chen H. Aurora B kinase as a novel molecular target for inhibition the growth of osteosarcoma. Mol Carcinog. (2019) 58:1056–67. doi: 10.1002/mc.22993 PMC6525060 30790360

[8] Chen S Li Y Zhi S Ding Z Wang W Peng Y. WTAP promotes osteosarcoma tumorigenesis by repressing HMBOX1 expression in an m(6)A-dependent manner. Cell Death Dis. (2020) 11:659. doi: 10.1038/s41419-020-02847-6 32814762 PMC7438489

[9] Sarkar S Bristow CA Dey P Rai K Perets R Ramirez-Cardenas A. PRKCI promotes immune suppression in ovarian cancer. Genes Dev. (2017) 31:1109–21. doi: 10.1101/gad.296640.117 PMC5538434 28698296

[10] Rehmani H Li Y Li T Padia R Calbay O Jin L. Addiction to protein kinase Cɩ due to PRKCI gene amplification can be exploited for an aptamer-based targeted therapy in ovarian cancer. Signal Transduct Target Ther. (2020) 5:140. doi: 10.1038/s41392-020-0197-8 32820156 PMC7441162

[11] Chen W Li Y Zhong J Wen G. circ-PRKCI targets miR-1294 and miR-186–5p by downregulating FOXK1 expression to suppress glycolysis in hepatocellular carcinoma. Mol Med Rep. (2021) 23:464. doi: 10.3892/mmr 33880589 PMC8097765

[12] Flum M Kleemann M Schneider H Weis B Fischer S Handrick R. miR-217–5p induces apoptosis by directly targeting PRKCI, BAG3, ITGAV and MAPK1 in colorectal cancer cells. J Cell Commun Signal. (2018) 12:451–66. doi: 10.1007/s12079-017-0410-x PMC5910322 28905214

[13] Moscat J Diaz-Meco MT. The interplay between PRKCI/PKCλ/ι, SQSTM1/p62, and autophagy orchestrates the oxidative metabolic response that drives liver cancer. Autophagy. (2020) 16:1915–7. doi: 10.1080/15548627.2020.1797290 PMC8386591 32686580

[14] Abdel-Moety A Baddour N Salem P El-Tobgy H El-Shendidi A. SQSTM1 expression in hepatocellular carcinoma and relation to tumor recurrence after radiofrequency ablation. J Clin Exp Hepatol. (2022) 12:774–84. doi: 10.1016/j.jceh.2021.12.001 PMC9168718 35677515

[15] Fujino T Goyama S Sugiura Y Inoue D Asada S Yamasaki S. Mutant ASXL1 induces age-related expansion of phenotypic hematopoietic stem cells through activation of Akt/mTOR pathway. Nat Commun. (2021) 12:1826. doi: 10.1038/s41467-021-22053-y 33758188 PMC7988019

[16] Guo JN Tian LY Liu WY Mu J Zhou D. Activation of the Akt/mTOR signaling pathway: A potential response to long-term neuronal loss in the hippocampus after sepsis. Neural Regener Res. (2017) 12:1832–42. doi: 10.4103/1673-5374.219044 PMC5745837 29239329

[17] Zhao H Zhang X Wang M Lin Y Zhou S. Stigmasterol simultaneously induces apoptosis and protective autophagy by inhibiting akt/mTOR pathway in gastric cancer cells. Front Oncol. (2021) 11:629008. doi: 10.3389/fonc.2021.629008 33708631 PMC7940753

[18] Liu Z Luo S Wu M Huang C Shi H Song X. LncRNA GHET1 promotes cervical cancer progression through regulating AKT/mTOR and Wnt/β-catenin signaling pathways. Biosci Rep. (2020) 40:BSR20191265. doi: 10.1042/BSR20191265 31682716 PMC6944656

[19] Cheng DD Li SJ Zhu B Yuan T Yang QC Fan CY. Downregulation of BZW2 inhibits osteosarcoma cell growth by inactivating the Akt/mTOR signaling pathway. Oncol Rep. (2017) 38:2116–22. doi: 10.3892/or.2017.5890 PMC5652953 28791373

[20] Xu D Qu L Hu J Li G Lv P Ma D. Transmembrane protein 106A is silenced by promoter region hypermethylation and suppresses gastric cancer growth by inducing apoptosis. J Cell Mol Med. (2014) 18:1655–66. doi: 10.1111/jcmm.12352 PMC4190911 24975047

[21] Qu L Li G Xia D Hongdu B Xu C Lin X. PRKCI negatively regulates autophagy via PIK3CA/AKT-MTOR signaling. Biochem Biophys Res Commun. (2016) 470:306–12. doi: 10.1016/j.bbrc.2016.01.059 26792725

[22] Gao W Guo H Niu M Zheng X Zhang Y Xue X. circPARD3 drives Malignant progression and chemoresistance of laryngeal squamous cell carcinoma by inhibiting autophagy through the PRKCI-Akt-mTOR pathway. Mol cancer. (2020) 19:166. doi: 10.1186/s12943-020-01279-2 33234130 PMC7686732

[23] Sadykova LR Ntekim AI Muyangwa-Semenova M Rutland CS Jeyapalan JN Blatt N. Epidemiology and risk factors of osteosarcoma. Cancer Invest. (2020) 38:259–69. doi: 10.1080/07357907.2020.1768401 32400205

[24] Moore DD Luu HH. Osteosarcoma. Cancer Treat Res. (2014) 162:65–92. doi: 10.1007/978-3-319-07323-1_4 25070231

[25] Harrison DJ Geller DS Gill JD Lewis VO Gorlick R. Current and future therapeutic approaches for osteosarcoma. Expert Rev Anticancer Ther. (2018) 18:39–50. doi: 10.1080/14737140.2018.1413939 29210294

[26] Kansara M Teng MW Smyth MJ Thomas DM. Translational biology of osteosarcoma. Nat Rev Cancer. (2014) 14:722–35. doi: 10.1038/nrc3838 25319867

[27] Lilienthal I Herold N. Targeting molecular mechanisms underlying treatment efficacy and resistance in osteosarcoma: A review of current and future strategies. Int J Mol Sci. (2020) 21:6885. doi: 10.3390/ijms21186885 32961800 PMC7555161

[28] Gill J Hingorani P Roth M Gorlick R. HER2-targeted therapy in osteosarcoma. Adv Exp Med Biol. (2020) 1257:55–66. doi: 10.1007/978-3-030-43032-0_5 32483730

[29] Kersting C Gebert C Agelopoulos K Schmidt H van Diest PJ Juergens H. Epidermal growth factor receptor expression in high-grade osteosarcomas is associated with a good clinical outcome. Clin Cancer Res. (2007) 13:2998–3005. doi: 10.1158/1078-0432.CCR-06-2432 17505002

[30] Bhattacharya B Home P Ganguly A Ray S Ghosh A Islam MR. Atypical protein kinase C iota (PKCλ/ι) ensures mammalian development by establishing the maternal-fetal exchange interface. Proc Natl Acad Sci U S A. (2020) 117:14280–91. doi: 10.1073/pnas.1920201117 PMC7322033 32513715

[31] Matsumoto M Ogawa W Akimoto K Inoue H Miyake K Furukawa K. PKClambda in liver mediates insulin-induced SREBP-1c expression and determines both hepatic lipid content and overall insulin sensitivity. J Clin Invest. (2003) 112:935–44. doi: 10.1172/JCI18816 PMC193669 12975478

[32] Kim KH Chung C Kim JM Lee D Cho SY Lee TH. Clinical significance of atypical protein kinase C (PKCι and PKCζ) and its relationship with yes-associated protein in lung adenocarcinoma. BMC Cancer. (2019) 19:804. doi: 10.1186/s12885-019-5992-7 31412817 PMC6693135

[33] Regala RP Davis RK Kunz A Khoor A Leitges M Fields AP. Atypical protein kinase C{iota} is required for bronchioalveolar stem cell expansion and lung tumorigenesis. Cancer Res. (2009) 69:7603–11. doi: 10.1158/0008-5472.CAN-09-2066 PMC2756303 19738040

[34] He M Wang G Jiang L Qiu C Li B Wang J. miR-486 suppresses the development of osteosarcoma by regulating PKC-δ pathway. Int J Oncol. (2017) 50:1590–600. doi: 10.3892/ijo.2017.3928 PMC5403184 28339053

[35] Ren L Hong SH Cassavaugh J Osborne T Chou AJ Kim SY. The actin-cytoskeleton linker protein ezrin is regulated during osteosarcoma metastasis by PKC. Oncogene. (2009) 28:792–802. doi: 10.1038/onc.2008.437 19060919 PMC7213760

[36] Hargarten JC Hu G Elsegeiny W Williamson PR. Measurement of SQSTM1 by flow cytometry. Autophagy. (2023) 19:2789–99. doi: 10.1080/15548627.2023.2224074 PMC10472860 37335017

[37] Grosjean I D’Andréa G Yazbeck N Belaid A Roméo B Domdom MA. Abstract 1284: The tumor scaffold protein SQSTM1 at the crossroads of DNA damage and immunotherapy responses in solid tumors. Cancer Res. (2022) 82:1284–. doi: 10.1158/1538-7445.AM2022-1284

[38] Zheng D Ning J Deng H Ruan Y Cheng F. TRIM26 inhibits clear cell renal cell carcinoma progression through destabilizing ETK and thus inactivation of AKT/mTOR signaling. J Trans Med. (2024) 22:481. doi: 10.1186/s12967-024-05273-w PMC11110379 38773612

[39] Fang S Luo Y Zhang Y Wang H Liu Q Li X. NTNG1 modulates cisplatin resistance in epithelial ovarian cancer cells via the GAS6/AXL/akt pathway. Front Cell Dev Biol. (2021) 9:652325. doi: 10.3389/fcell.2021.652325 34277602 PMC8281315

[40] Sun X Shi C Dai J Zhang MQ Pei DS Yang L. Targeting the mitochondrial protein YME1L to inhibit osteosarcoma cell growth in *vitro* and in *vivo* . Cell Death Dis. (2024) 15:346. doi: 10.1038/s41419-024-06722-6 38769124 PMC11106333

[41] Tewari D Patni P Bishayee A Sah AN Bishayee A. Natural products targeting the PI3K-Akt-mTOR signaling pathway in cancer: A novel therapeutic strategy. Semin Cancer Biol. (2022) 80:1–17. doi: 10.1016/j.semcancer.2019.12.008 31866476

[42] Ji Z Shen J Lan Y Yi Q Liu H. Targeting signaling pathways in osteosarcoma: Mechanisms and clinical studies. MedComm. (2023) 4:e308. doi: 10.1002/mco2.308 37441462 PMC10333890

[43] Cheng DD Li SJ Zhu B Zhou SM Yang QC. EEF1D overexpression promotes osteosarcoma cell proliferation by facilitating Akt-mTOR and Akt-bad signaling. J Exp Clin Cancer Res. (2018) 37:50. doi: 10.1186/s13046-018-0715-5 29510727 PMC5839064

[44] Zhu YR Zhou XZ Zhu LQ Yao C Fang JF Zhou F. The anti-cancer activity of the mTORC1/2 dual inhibitor XL388 in preclinical osteosarcoma models. Oncotarget. (2016) 7:49527–38. doi: 10.18632/oncotarget.v7i31 PMC5226526 27385099

[45] Wang X Song H Fang L Wu T. EIF4A3-mediated circPRKCI expression promotes triple-negative breast cancer progression by regulating WBP2 and PI3K/AKT signaling pathway. Cell Death discovery. (2022) 8:92. doi: 10.1038/s41420-022-00892-y 35236829 PMC8891274

[46] Abdelatty A Fang D Wei G Wu F Zhang C Xu H. PKCι Is a promising prognosis biomarker and therapeutic target for pancreatic cancer. Pathobiol: J immunopathol Mol Cell Biol. (2022) 89:370–81. doi: 10.1159/000521588 35785767

[47] Li S Zhang H Shang G. Current status and future challenges of CAR-T cell therapy for osteosarcoma. Front Immunol. (2023) 14:1290762. doi: 10.3389/fimmu.2023.1290762 38187386 PMC10766856

