# Correction: Downregulation of PRKCI inhibits osteosarcoma cell growth by inactivating the Akt/mTOR signaling pathway

**DOI:** 10.3389/fonc.2025.1643772

**Published:** 2025-07-07

**Authors:** Liujing Qu, Yu Xin, Jieni Feng, Xiaolei Ren, Zuming Li, Xueru Chen, Guangyan Miao, Jiankun Chen, Chengming Sun, Yue Lu

**Affiliations:** ^1^ Department of Clinical Laboratory, The Affiliated Yantai Yuhuangding Hospital of Qingdao University, Yantai, China; ^2^ Department of Medical Laboratory, Qingdao Sixth People’s Hospital, Qingdao, China; ^3^ The Second Clinical Medical College, Guangzhou University of Chinese Medicine, Guangzhou, China; ^4^ Department of Molecular, Cell and Cancer Biology, University of Massachusetts Chan Medical School, Worcester, MA, United States; ^5^ The Third Comprehensive Department, The Second Affiliated Hospital of Guangzhou University of Chinese Medicine (Guangdong Provincial Hospital of Chinese Medicine), Guangzhou, China; ^6^ State Key Laboratory of Dampness Syndrome of Chinese Medicine, The Second Affiliated Hospital of Guangzhou University of Chinese Medicine (Guangdong Provincial Hospital of Chinese Medicine), Guangzhou, China; ^7^ Guangdong-Hong Kong-Macau Joint Lab on Chinese Medicine and Immune Disease Research, Guangzhou University of Chinese Medicine, Guangzhou, China

**Keywords:** PRKCI, osteosarcoma, proliferation, Akt/mTOR signaling pathway, therapy

In the published article, there was an error in **Figure 5A** and **Figure 6A** as published. During an AI-assisted image integrity review of our published work, we identified inadvertent duplications in **Figure 5A** and **Figure 6A** (Migration, PRKCI and shcontrol; Wound healing assay, 0h shcontrol and shPRKCI, 24h Vector and shPRKCI). This was an oversight during figure preparation and does not affect the study’s conclusions. We have since located the original data and prepared a corrected version of the figure. The corrected **Figure 5A** and **Figure 6A** and its caption appear below.

**Figure 5:**
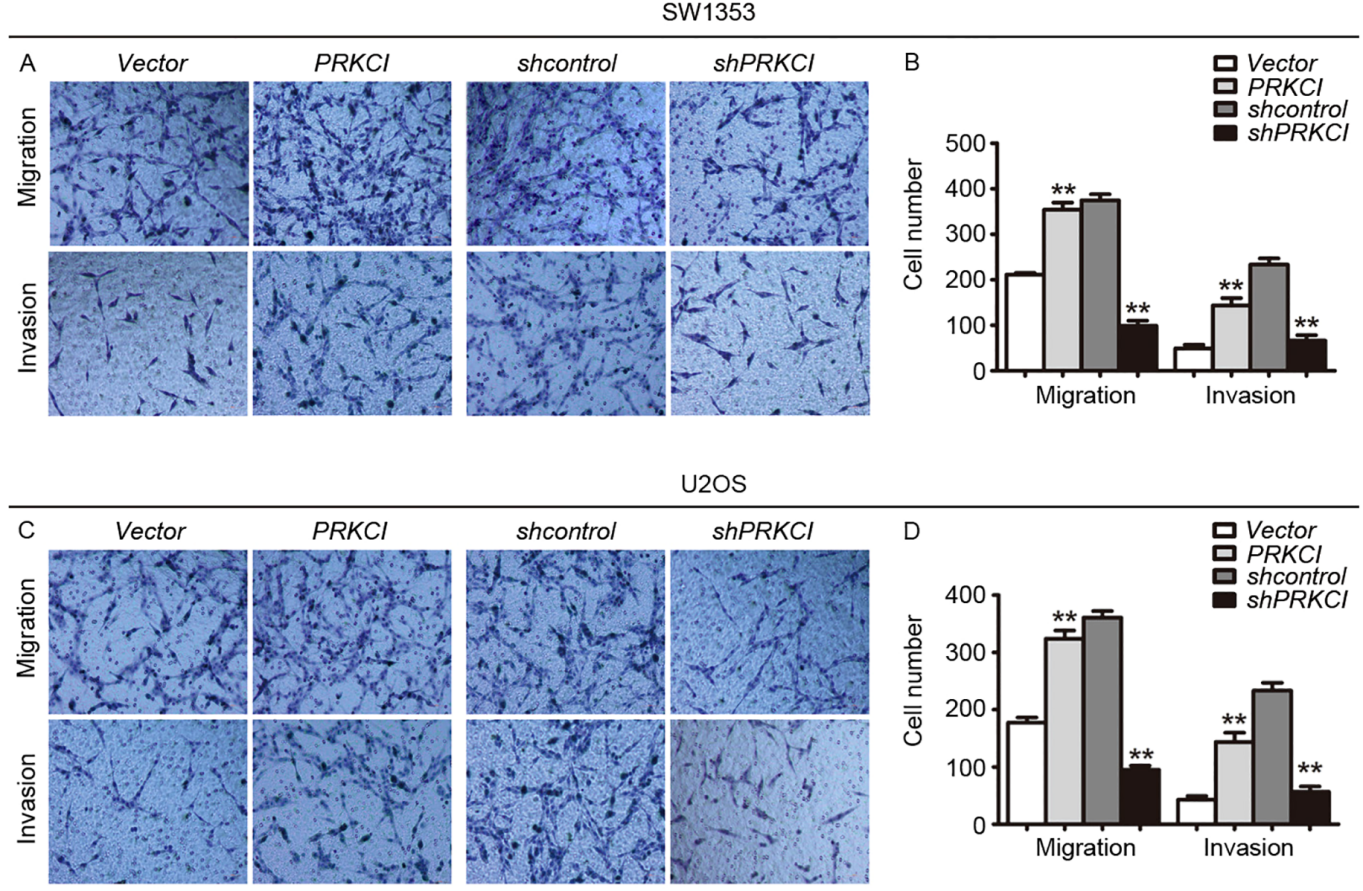
The Transwell system was used to evaluate the effect of PRKCI on the migration and invasion of osteosarcoma cells. **(A, C)** Transwell migration assay and Matrigel invasion assay for SW1353 cells or U2OS cells after transfection empty vector or PRKCI plasmid for 24 h (shcontrol or shPRKCI for 48 h). Cells were stained with crystal violet (magnification: ×200). **(B, D)** Quantification of invaded and migrated SW1353 cells or U2OS cells. (Data were based on three independent experiments and shown as the mean ± SEM, **p < 0.01).

**Figure 6:**
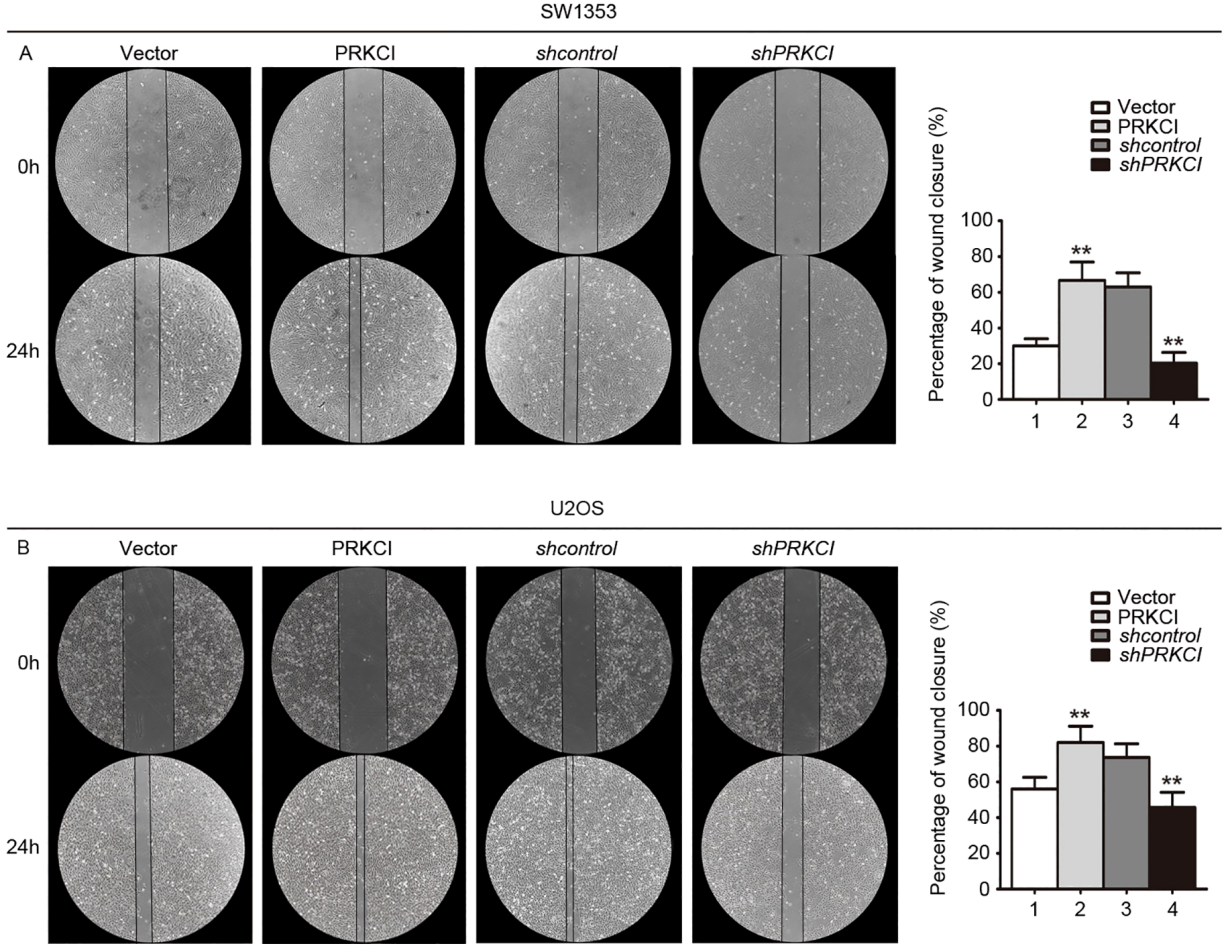
A wound-healing assay was used to evaluate the effect of PRKCI on the migration of osteosarcoma cells. **(A, B)** Microscopic images of wound-healing assay data for SW1353 and U2OS cells transfected with empty vector or PRKCI plasmid for 24 h (shcontrol or shPRKCI for 48 h). (Data were based on three independent experiments and shown as the mean ± SEM, **p < 0.01).

The authors apologize for this error and state that this does not change the scientific conclusions of the article in any way. The original article has been updated.

